# Precision Medicine in Neonates: Future Perspectives for the Lung

**DOI:** 10.3389/fped.2020.586061

**Published:** 2020-10-30

**Authors:** Wes Onland, Jeroen Hutten, Martijn Miedema, Lieuwe D. Bos, Paul Brinkman, Anke H. Maitland-van der Zee, Anton H. van Kaam

**Affiliations:** ^1^Department of Neonatology, Amsterdam University Medical Centers, VU University Medical Center, Emma Children's Hospital, University of Amsterdam, Amsterdam, Netherlands; ^2^Department of Respiratory Medicine, Amsterdam University Medical Centers, University of Amsterdam, Amsterdam, Netherlands

**Keywords:** individualized medicine, targeted treatment, personalized medicine, newborn, neonatal intensive care

## Abstract

Bronchopulmonary dysplasia (BPD) is the most common complication of pre-term birth with long lasting sequelae. Since its first description more than 50 years ago, many large randomized controlled trials have been conducted, aiming to improve evidence-based knowledge on the optimal strategies to prevent and treat BPD. However, most of these intervention studies have been performed on a population level without regard for the variation in clinical and biological diversity (e.g., gestational age, ethnicity, gender, or disease progression) between patients that is driven by the complex interaction of genetic pre-disposition and environmental exposures. Nevertheless, clinicians provide daily care such as lung protective interventions on an individual basis every day despite the fact that research supporting individualized or precision medicine for monitoring or treating pre-term lungs is immature. This narrative review summarizes four potential developments in pulmonary research that might facilitate the process of individualizing lung protective interventions to prevent development of BPD. Electrical impedance tomography and electromyography of the diaphragm are bedside monitoring tools to assess regional changes in lung volume and ventilation and spontaneous breathing effort, respectively. These non-invasive tools allow a more individualized optimization of invasive and non-invasive respiratory support. Investigation of the genomic variation in caffeine metabolism in pre-term infants can be used to optimize and individualize caffeine dosing regimens. Finally, volatile organic compound analysis in exhaled breath might accurately predict BPD at an early stage of the disease, enabling clinicians to initiate preventive strategies for BPD on an individual basis. Before these suggested diagnostic or monitoring tools can be implemented in daily practice and improve individualized patient care, future research should address and overcome their technical difficulties, perform extensive external validation and show their additional value in preventing BPD.

## Introduction

Improvements in neonatal care have led to an increased survival of very low birth weight (VLBW) infants over the past decades (1, 2). Evidence based intervention aiming to improve the pulmonary condition, such as exogenous surfactant treatment, antenatal corticosteroids, more gentle modes and restrictive use of invasive ventilation have greatly contributed to this improved survival (3–5). However, many of the VLBW infants will be at high risk of developing bronchopulmonary dysplasia (BPD), which is considered the most common complication after pre-term birth (6). BPD is histologically characterized by an arrest in normal lung development, resulting in a prolonged need for respiratory support and (re)hospitalization (7). Although studies have consistently shown the independent association between BPD and increased risk for repeated respiratory infections (bacterial and viral), asthma, and a compromised lung function lasting into adolescence (7, 8), not every infants with the diagnosis BPD will suffer from these pulmonary sequelae. However, there is also a general concern that pre-term infants with BPD have more risk of developing chronic obstructive pulmonary disease in later life (9). In addition to pulmonary sequelae, BPD is also associated with an increased risk of cerebral palsy and developmental delay (10). BPD is considered a multifactorial disease with genetic susceptibility, intrauterine growth restriction, nutritional deficits, oxygen toxicity, pulmonary inflammation, and direct mechanical injury caused by mechanical ventilation as the most important risk factors (11–13).

Over the last decades, several interventions aiming to reduce the incidence of BPD have been studied in large high quality randomized controlled trails, but the results have so far been disappointing. In contrast to other morbidities related to pre-term birth, the incidence of BPD as not declined over time (2). It is important to acknowledge that most intervention studies, although restricting eligible participants to infants below 30 weeks of gestational age, used a population based approach, targeting infants based on a single, often indirect, risk factor for BPD, such as having a gestational age below 30 weeks gestational age or treatment with invasive mechanical ventilation. This approach does not account for the complexity of developing BPD, and the individual diversity that is often present in infants randomized in these studies. At an individual level, a pre-term infant might have to be differentiated into different respiratory disease phenotypes using for instance biomarkers, metabolomics, and genomics (14). However, in order to prevent pre-mature phenotyping based on clinical intuition, the hypothesis of multiple BPD phenotypes needs to be investigated extensively using large subgroups of datasets after external validation before it can be used in neonatal precision medicine of the lung (15). Using a similar line of reasoning, the intervention applied should probably be tailored to the individual patient and lung characteristics instead of using the “one size fits all” approach routinely investigated in large randomized controlled trials (16). This individualized approach using both prognostic enrichment or predictive enrichment in randomized controlled trials requires individual monitoring of the BPD risk profile, the underlying respiratory phenotype, and the correct application of the intervention (17, 18).

Precision medicine, also referred to as personalized or individualized medicine has become an increasingly used approach in adult and pediatric research (19). Prognostic enrichment in precision medicine focusses on patient stratification based on a combination of clinical (e.g., gestational age, ethnicity, gender) information, genetic pre-disposition, and individual biomarkers, enabling more precise differentiation of different phenotypes within this group of pre-term infants (20), whereas predictive enrichment identifies subgroups of patients with a higher chance of responding to a specific therapy based on a specific clinical or biological phenotype (18). However, a literature search in MEDLINE combining the words “precision” or “individualized” and “pulmonary” and “pre-term” results in zero citations. This suggests that individualized risk profiling and tailored intervention for BPD, are not part of daily practice and/or research. This is largely caused by lack of appropriate monitoring tools to assess the individual risk of developing BPD and the successful and correct application of intervention to reduce BPD. This narrative review highlights some promising developments in neonatal pulmonary research enabling the use of precision medicine in the search for more effective interventions to prevent BPD.

## Electrical Impedance Tomography

As previously mentioned, invasive mechanical ventilation is one of the risk factors for developing BDP. Pre-clinical studies have indicated that overdistension (volutrauma) and collapse (atelectrauma) of alveoli play a major role in ventilator induced lung injury and subsequent development of BPD. Pre-term infants are prone to loss of lung volume due to their immature lung physiology and underlying lung disease. This leads to impaired lung function and respiratory failure and the need for non-invasive or invasive respiratory support to restore gas exchange. Ideally, respiratory support should reverse atelectasis and avoid alveolar overdistention, resulting in homogeneous aeration and ventilation of the lungs. Reaching this goal requires an individual and dynamic approach, as lung condition differs between patients and lung disease is often heterogeneous in nature. This heterogeneity results in co-existence of overdistended and collapsed lung regions in the same lung (21, 22). It is clear that monitoring of regional aeration and ventilation is essential to individualize respiratory support in pre-term infants with respiratory failure and at risk for developing BPD.

Monitoring regional ventilation distribution and end expiratory lung volume in pre-term infants is challenging, especially at the bedside. Currently available monitoring tools such as chest X-ray, tracer gas wash-in/out methods, or respiratory inductive plethysmography have serious limitations, including lack of regional and online information, cumbersome to perform at the bedside, and use of radiation. Electrical Impedance Tomography (EIT) is a technique developed in the early 1980's that uses differences in tissue conductance in response to an electrical currents to visualize changes in lung aeration (23). EIT is non-invasive, radiation free, and generates continuous bedside information on relative changes in regional ventilation distribution and end expiratory lung volume, which shows a high correlation to actual intra-thoracic changes in air-content (24). Measurement requires placement of a belt containing non-sticky electrodes placed around the chest at the level of the nipple.

**Figure 1:**
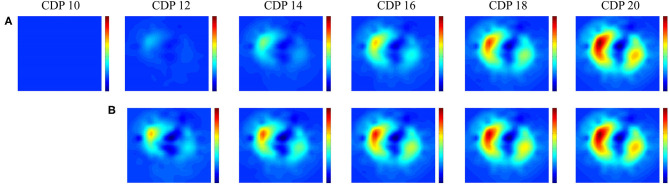
Lung recruitment visualized by Electric Impedance Tomography. Change in functional electrical impedance tomography in end expiratory lung impedance during an oxygenation guided lung recruitment procedure in a high frequency ventilated pre-term infant (945 grams). The impedance changes are referenced to the starting pressure of 8 cm H_2_O. Row **(A)** shows the inflation and row **(B)** the deflation limb of the recruitment procedure (39). All images have the same scale where red indicates a large change and blue a small change in impedance.

In pre-term infants, EIT research has mainly focused on the pathophysiology of lung disease and the impact of interventions on (regional) lung aeration. Studies have shown that EIT is able to detect and monitor changes in (regional) aeration caused by pneumothoraces (25–27), postural changes (28, 29), atelectasis (30), incorrect endotracheal tube placement (31), endotracheal suctioning (32, 33), (minimal) invasive surfactant administration (34, 35), changes in nasal continuous positive pressure levels (36, 37), and lung recruitment procedures during conventional and high frequency ventilation in pre-term infants (Figure 1) (38, 39).

These observational studies show the potential of EIT to individualize respiratory care in pre-term infants at high risk of developing BPD. EIT can assist the clinician in the challenge to optimize ventilator support at an individual level, and thereby achieving the goal of homogeneous non-injurious ventilation. Although EIT has important potential, there are still some technical and practical issues that need to be resolved before it can be implemented in clinical practice. First, results from most studies are based on off-line EIT data analysis. Software allowing continuous online analysis of EIT data is currently being developed. Second, well-designed easily applicable equipment for neonates is still lacking. A user-friendly textile electrode belt, a well-tolerated skin conductance substance, and wireless recording are needed for successful implementation in clinical practice. These improvements will also allow testing of a longer recording time than what was used in most observational studies (1–2 h). Third, developmental and production costs of the hardware need to be reduced in order to make implementation feasible from an economic perspective. Finally, the effect of EIT on important clinical outcomes needs to be investigated in future studies.

### Key Messages

- EIT can visualize regional lung volume and ventilation changes at the bedside and individualize pulmonary treatment.
- Future development should focus on improvement in EIT hardware and software so the final step to clinical implementation can made.

## Electromyography of The Diaphragm

**Figure 2:**
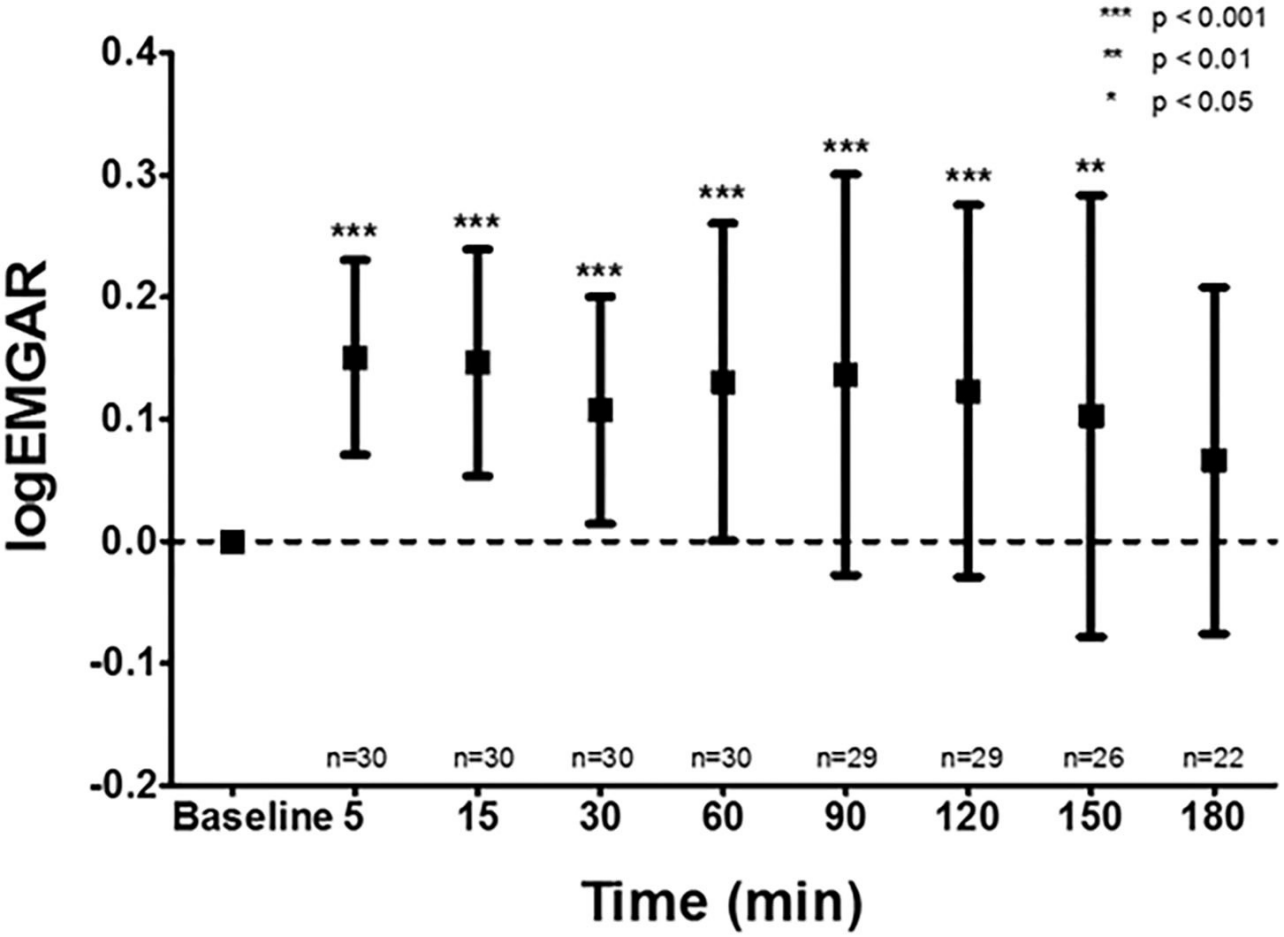
Effect of caffeine on the amplitude of the diaphragm measured by electromyography. The transcutaneous electromyographic (EMG) analysis of the diaphragm showed a significant increase in logarithm of the EMG Activity Ratio (logEMGAR) after a loading dose caffeine, compared to baseline. The logEMGAR described the relative changes in EMG activity, either increasing or decreasing symmetrical around unity.

Respiratory failure is a common complication in pre-term infants primarily caused by an impaired control of breathing and a compromised lung function (40). Impaired control of breathing leads to apnea of pre-maturity, which can lead to hypoxemia and bradycardia, thereby increasing the risk of adverse neurodevelopmental outcome (41). In an attempt to stabilize the respiratory system and reduce the work of breathing, pre-term infants often receive respiratory support. Non-invasive support is the preferred modality, which can be applied via continuous nasal positive airway pressure (nCPAP), heated humidified high flow nasal cannula, and nasal intermittent positive pressure ventilation (nIPPV) (42). If this is insufficient to restore gas exchange and work of breathing, endotracheal intubation and invasive mechanical ventilation may be necessary. Despite the frequent use of respiratory support, objective criteria to select the optimal mode and setting are not well-established (43, 44). Conventional parameters such as oxygen need and blood gas analysis are not very specific and lack information on work of breathing. As a result, respiratory support modes are often selected and set according to general protocols and timely individual titration is often lacking. This might have important consequences, because both too little and too much support may injure the lungs. In case non-invasive support is adjusted, most clinicians use a “trial and error” strategy (43). For example, the nCPAP pressure is lowered and if the patients' respiratory condition deteriorates, the pressure is increased to the previous setting. Ideally, selection and weaning off the mode and level of respiratory support should be based on bedside, continuous and quantitative individual information on breathing activity or the work of breathing. As the diaphragm is the main respiratory muscle in pre-term infants, retrieving information on its activity might provide objective information on breathing activity. Electrical activity of the diaphragm can be with electromyography (dEMG) and there are currently two methods to detect the electrical signal of the diaphragm; the transcutaneous method in which sensors are placed on the skin and the invasive transesophageal method in which sensors are mounted on a catheter positioned in the esophagus. Recent studies have shown that both techniques are feasible in pre-term infants (45, 46), and are able to detect changes in diaphragmatic activity (47, 48) (Figure 2). However, transesophageal dEMG is relatively invasive, expensive and only available on one specific ventilator. The transcutaneous method is less invasive, cheap, and uses stand-alone equipment allowing its use during all modes of respiratory support, independent of the ventilator. Observational studies in pre-term infants have shown that transcutaneous dEMG is able to detect changes in diaphragmatic activity in response to weaning the mode of respiratory support from nCPAP to low flow nasal cannula. Furthermore, the diaphragmatic activity was significantly higher in those infants that failed this transition compared to those in who weaning was successful (49).

In addition to unloading the additional work of breathing, the delivered respiratory support should be, if applicable, synchronized to the individual breathing efforts of the infants. Historically, changes in airway pressure or flow are used for synchronization of invasive mechanical ventilation (50). However, these parameters are not always accurate in the presence of leak, either around the endotracheal tube or via the upper airways during nasal positive pressure ventilation (51). Being independent of airway flow or pressure, dEMG might therefore also be an ideal candidate for triggering respiratory support. Indeed, measuring the electrical activity of the diaphragm by the transesophageal method can be used for synchronizing the individual breaths with the ventilator (52). A Cochrane review performed in 2017 identified one small underpowered randomized control trial comparing conventional ventilation to ventilation triggered by diaphragmatic activity with no significant effects on the primary outcomes. However, the conclusion of that review was that lower level evidence studies, such as case series and non-randomized cross over studies are suggesting a physiological benefit associated with diaphragma triggered ventilators, and that new high quality randomized controlled studies are urgently needed (53).

Studies have also shown that (spontaneous) breath detection is feasible and appropriate via transcutaneous dEMG (54). Optimal triggering might also have clinical implications, as studies suggest that synchronized nasal positive pressure ventilation reduces the need for invasive mechanical ventilation (55).

It is clear that measuring diaphragmatic activity with dEMG has the potential to individualize the application of respiratory support in pre-term infants. However, more and larger studies are needed to determine which dEMG output parameters provide the best information on the individual patients' needs. Furthermore, transcutaneous dEMG triggering of non-invasive support still needs to be tested in pre-term infants.

### Key Messages

- Most clinicians use a “trial and error” strategy when selecting and setting the mode of respiratory support. Furthermore, synchronizing support to the individual spontaneous breathing effort is often not optimal, especially during non-invasive support.
- The activity of the diaphragm can be measure with electromyography and provides objective information on the patients' breathing effort. Furthermore, breath detection is feasible and accurate with dEMG.
- dEMG can potentially be used to titrate and trigger the mode and level of respiratory support but future studies are needed to explore these potential indications in pre-term infants.

## Pharmacogenetics and Caffeine

Administration of caffeine to pre-term infants is standard of care in all neonatal intensive care units. A large randomized controlled trial provided solid evidence that besides reducing apnea of pre-maturity (56), pre-term infants treated with caffeine have a reduced risk of BPD (57), and an improved neurodevelopmental outcome at 2 years corrected age (58, 59). Despite this clear evidence, there are uncertainties regarding the exact working mechanism of caffeine and the optimal caffeine dose (60). Comparable to the adult population, effective and safe dosing probably differs between pre-term infants (61). Standardized regimens, i.e., the same dose to all infants, lead to high variation of serum levels of caffeine and its metabolites (62). This observation is important because it has been shown that in some infants a high caffeine loading-dose is associated with negative effects on pre-term infant brain development (63). On the other hand, low caffeine concentrations may lead to insufficient apnea treatment, increased use of invasive mechanical ventilation posing a higher risk for BPD and neurodevelopmental impairment (64). Since most studies evaluated the effect of a standardized caffeine dosage regimen on important neonatal outcomes at population level, they were neither able to determine patient-specific risks and benefits nor incorporate these in the evaluation of an optimal dosing regimen. Heterogeneity due to genomic differences likely contributes to the lack of a strong correlation between serum caffeine levels and clinical effects. Precision medicine might help to optimize caffeine dosing and treatment-associated outcomes. Caffeine metabolism is limited in pre-term infants because cytochrome P4501A2 (CYP1A2) enzyme activity is markedly reduced relative to term neonates, leading to a prolonged half-life and increased urinary excretion of unmetabolized caffeine. Enzyme activity increases with increasing gestational age and advancing post-natal age (65). Although the exact molecular mechanisms underlying the benefits of caffeine administration have not been elucidated (57), it is likely that genomic and metabolomic heterogeneity influences optimal patient dose due to infant-specific caffeine metabolism and risk susceptibility (66–68). Several candidate genes have been suggested based on the known genetic associations with caffeine (e.g., cytochrome P450 enzymes, adenosine receptors) (66, 69–72).

It is highly likely that there is a dose-specific response to caffeine treatment in pre-term neonates. Individual genomic variants are a potential indicator for its effectiveness but also the risk to develop complications due to caffeine treatment. Genomic variance probably explains why the correlation between caffeine plasma concentrations and clinical outcome remains poor. An observational cohort study including infants with a gestational age ≤ 30-weeks and treated with caffeine should be conducted to investigate to which extent genomic variation contributes to this poor correlation, and to what extend adopting individualize treatment in this population can improve outcome. In the future, clinicians may be able to evaluate a genomic profile at birth which they can then utilize to determine a personalized caffeine dose.

### Key Messages

- Caffeine treatment is a well-established evidence-based standard of care treatment for the prevention of BPD.
- Similar to adults, pre-term infants might need individual dosing of caffeine based on their genomic profile.
- Future research should investigate the correlation between genomic variation in caffeine metabolism to optimize and individualize caffeine treatment in pre-term infants.

## Volatile Organic Compounds

Many interventions to reduce the risk of BPD have been tested in randomized clinical trials, but to date few have shown to be effective and safe. It has been suggested that these disappointing results might be caused by failure of clinical variables or biomarkers to accurately predict the risk of BPD at an early stage in life. This is also called a lack of prognostic enrichment: the study population has a low a-priori risk for the outcome of interest and therefore many patients are exposed to the treatment without any chance of a positive effect, limiting efficacy, yet experiencing the side effects, increasing safety concerns (73). Consistent with this assumption, a systematic review summarizing all published clinical prediction models failed to show accuracy in discriminating and calibrating performance after external validation using a large individual patient database (74). Therefore, clinical prediction models are infrequently used in current clinical practice and research.

Besides prognostic enrichment, trials may further benefit from predictive enrichment: increasing the likelihood of the included patient for a beneficial treatment response. Although BPD is multifactorial in nature, inflammation and growth failure are considered important risk factors and mediators in its development. Most BPD associated inflammatory and growth factor biomarkers investigated, such as interleukin-6 or−8, monocyte chemoattractant protein-1, vascular endothelial growth factor, keratinocyte growth factor, angiopoietin 2 and interferon-γ failed as predictors of BPD development (75) emphasizing the complex pathophysiology. Irrespective of this underwhelming result for BPD prediction, a set of biomarkers that identifies a homogeneous group of patients with a shared pathogenesis might serve as a predictive tool for treatment response. However, it is important to emphasize that most evaluated biomarkers require sampling of urine, blood or saliva, and need complicated laboratory analysis techniques. This may hamper implementation in clinical practice as these techniques are usually not available in every hospital. This means that the search for better predictive indices with bedside availability, without the need for complicated laboratory techniques, and preferably without requiring blood needs to be continued.

Exhaled breath might be the medium that meets the requirements for such a prognostic and predictive test in pre-term infants. Collection is fully non-invasive and analysis may be rapid when sensor technology is used. Hundreds to thousands of volatile organic compounds (VOCs) have been described in exhaled breath, which represent metabolic processes in the host, bacterial metabolism, and organ function (76). VOCs are reported to serve as potential biomarker in several adult respiratory diseases, such as pleural mesothelioma, pulmonary sarcoidosis, asthma, chronic obstructive pulmonary disease, ventilator associated pneumonia and acute respiratory distress syndrome (77–79).

There are several distinct technologies to analyze exhaled breath of which gas chromatography-mass spectrometry (GC-MS) and electronic nose analysis (eNose) are most frequently used. VOCs can be separated, quantified and identified by GC-MS, which remains the gold-standard for untargeted biomarker analysis in exhaled breath. GC-MS analysis is a time-consuming and off-line analysis, making it unpractical as a clinical prediction instrument. However, it does enable researchers to measure a broad range of compounds and identify unknown compounds semi-quantitatively. A diagnostic study that uses GC-MS for breath analysis will therefore result in a list of potential biomarkers. These potential markers can be used to develop a disease-specific measurement tool based on technologies that can provide rapid, bedside results. One technique that has gained substantial traction is the eNose, which relies of on sensor technology (79). The eNose enables real-time analyses of the patterns of selected VOCs in complex gas mixtures. It does not allow measurement of individual VOCs, but uses pattern recognition to capture composite VOC mixtures by cross-reactive sensors, called a breath-print (80). This device is highly attractive in daily clinical practice since it can be used at the bedside and because it provides instant results, which is so highly needed in predicting BPD at an early age.

Given the multifactorial etiology of BPD, analysis of exhaled breath of pre-term infants with GC-MS might enable us to quantify the prognostic accuracy of individual and combinations of VOCs in exhaled breath. As BPD has several pathophysiological links to lung injury in adults, we expect that the VOCs might also be equipped to detect markers of BPD. One of the challenges will be the collection of breath in pre-term infants. The minute volume ventilation is quite low and there is a relatively high bias flow delivered by the devices used for respiratory support.

**Figure 3:**
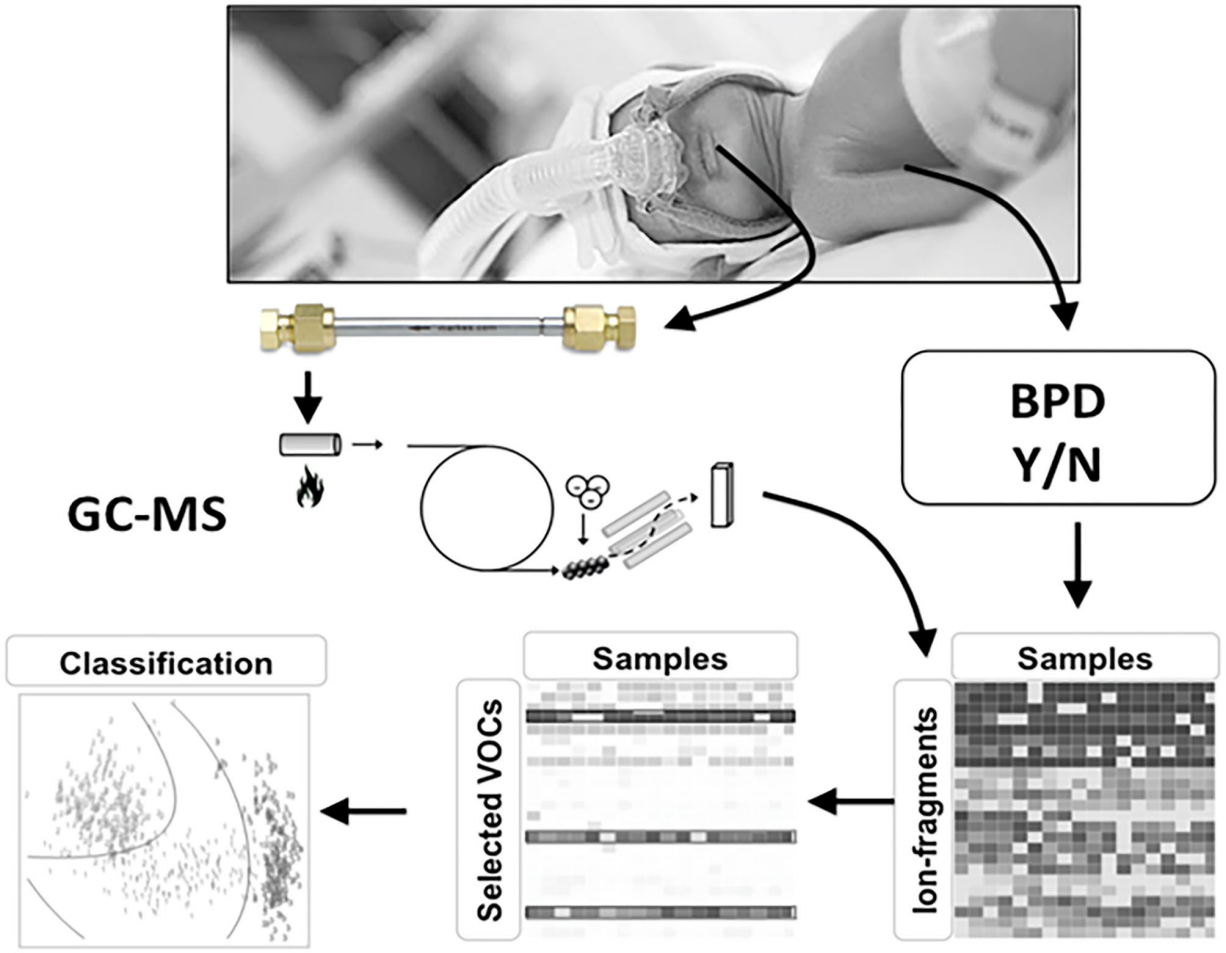
Study design for developing BPD specific eNose. Volatile organic compounds (VOCs) are absorbed onto a stainless-steel tube containing Tenax GR 60/80 (Interscience, Breda, The Netherlands) for 5 min at a flow rate of 50 ml/min. The captured VOCs in the tubes are released by re-heating the tubes after which the fragment ions are detected using a quadrupole mass spectrometer (GCMS-GP2010; Shimadzu, Den Bosch, The Netherlands) with a scan range of 37–300 Da. Ion fragment peaks were used for statistical analysis. GC-MS analysis will be performed in all infants.

A recent study suggested that the eNose can discriminate pre-term infants developing BPD at an early age from those who do not. However, this study only included mechanically ventilated infants, using headspace analysis of tracheal aspirates rather than exhaled breath (81). Nowadays, more and more infants are initially managed without invasive ventilation and tracheal aspirates are therefore not available in these infants (4). We studied breaths of four pre-term infants on non-invasive respiratory support in a pilot study. Several breath collection techniques were investigated, but the introduction of a suction catheter positioned under the nasal mask was the only technique that provided positive results defined as a range of known human VOCs above the detection limit. We are currently collecting exhaled breaths at multiple time points in 100 pre-term infants born <28 weeks of gestation, treated with both invasive and non-invasive respiratory support (Figure 3). The GC-MS analyses will be performed, allowing us to determine which selected set of VOCs to focus on when developing the eNose for identifying infants at risk of BPD within the first week of life. Once the GC-MS analyses of exhaled breath in pre-term infants show which selected VOCs predict BPD, an eNose specific breath print for BPD can be developed following an efficient translation from promising biomarker to established bedside tool for predicting BPD as described in the BEST (Biomarkers, EndpointS, and other Tools) Resource (82). This has to be validated in a large population of pre-term infants in a multicenter setting. After showing a good external validation, the final step will be performing an impact analysis showing the additional value of this prediction model. Another potential application of exhaled breath analysis might be discriminating between different (main) pathophysiological causes of BPD. An appropriate grown pre-term infant born after severe chorioamnionitis might develop a different BPD phenotype than a pre-term infants born after severe intrauterine growth retardation. If the development of the eNose shows promising discrimination between infants with and without the development of BPD, it might also be possible to differentiate in infants with BPD what is the main pathophysiological pathway is and select the optimal treatment accordingly.

### Key Messages

- To date, there is no prediction model based on clinical characteristics or biomarkers with accurate discriminating ability to detect BPD at an early stage.
- Measuring VOCs is increasingly being used in adult respiratory medicine.
- Observational cohort studies should investigate which VOCs in exhaled breath accurately predict BPD at an early stage of the disease.

## Limitations

First, given the narrative nature of the article, this review has the limitation that we did not identify systematically all publications regarding precision medicine for pre-term lungs. We have selected these four items on precision medicine because the recent publication dates, and are deemed most promising to have an important clinical impact. Second, every of these promising technological, diagnostic or prognostic tools need further development to go from bench to bedside tools, need extensive internal and external validation before submitted to impact analyses in daily practice.

## Conclusion

These four presented potential developments in pulmonary research might improve precision medicine in order to prevent development of BPD. EIT and dEMG are bedside monitoring tools with the potential to individualize invasive and non-invasive respiratory support, whereas pharmacogenetic research of caffeine metabolism and VOC analysis of exhaled breath might optimize preventive drug therapy for BPD on an individual basis.

## Author Contributions

MM and AVK had specific focus on the section on electrical impedance tomography. WO and AHM-vdZ had a specific focus on the section of pharmacogenetics and caffeine. WO, PB, and LB had a specific focus on the section of volatile organic compounds. JH and AVK had a specific focus on the section of electromyography. All authors provided crucial pulmonary expertise on these sections and the introduction. All authors approved the final version of the paper.

## Conflict of Interest

WO was funded by the Chiesi onlus foundation. LB was supported via the Dutch Lung Foundation (Young Investigator Grant, Dirkje Postma Award and Industry-Academia-Partnership) and via the IMI. PB was supported via the Amsterdam UMC Innovation Grant. AM-vdZ reported unrestricted research grants from GSK, Boehringer Ingelheim and Vertex and personal fees paid to the university from GSK, Boehringer Ingelheim, and Astra Zeneca for participating in advisory boards and lecturing at symposia, outside the submitted work. AVK had received grants from Chiesi Pharmaceuticals and Vyaire Medical. The remaining authors declare that this research was conducted in the absence of any commercial or financial relationships that could be construed or perceived as a potential conflict of interest.
